# Fungal footprints in oral cancer: unveiling the oral mycobiome

**DOI:** 10.3389/froh.2024.1360340

**Published:** 2024-03-14

**Authors:** Jessica Sonal Monteiro, Kriti Kaushik, José Alcides Almeida de Arruda, Eleni Georgakopoulou, Angelica Thomaz Vieira, Tarcilia A. Silva, Darshana Devadiga, Charles E. Anyanechi, Sameep Shetty

**Affiliations:** ^1^ Department of Oral and Maxillofacial Surgery, Manipal College of Dental Sciences Mangalore, Manipal Academy of Higher Education, Manipal, India; ^2^ Department of Oral and Maxillofacial Surgery, Manipal College of Dental Sciences, Mangalore, India; ^3^ Department of Oral Diagnosis and Pathology, School of Dentistry, Universidade Federal do Rio de Janeiro, Rio de Janeiro, Brazil; ^4^ Laboratory of Histology-Embryology, Molecular Carcinogenesis Group, Medical School, National and Kapodistrian University of Athens, Athens, Greece; ^5^ Laboratory of Microbiota and Immunomodulation, Department of Biochemistry and Immunology, Institute of Biological Sciences, Universidade Federal de Minas Gerais, Belo Horizonte, Brazil; ^6^ Department of Oral Surgery, Pathology and Clinical Dentistry, School of Dentistry, Universidade Federal de Minas Gerais, Belo Horizonte, Brazil; ^7^ Department of Conservative Dentistry and Endodontics, AB Shetty Memorial Institute of Dental Sciences, NITTE (Deemed to be University), Mangalore, India; ^8^ Department of Oral and Maxillofacial Surgery, University of Calabar/University of Calabar Teaching Hospital, Calabar, Nigeria

**Keywords:** fungi, microbiota, mycobiome, oral cancer, oral mucosa, oral oncology

## Abstract

Oral squamous cell carcinoma (OSCC) is the most common type of head and neck cancer, with a high mortality rate. There is growing evidence supporting a link between oral cancer and the microbiome. The microbiome can impact various aspects of cancer, such as pathogenesis, diagnosis, treatment, and prognosis. While there is existing information on bacteria and its connection to oral cancer, the fungi residing in the oral cavity represent a significant component of the microbiome that remains in its early stages of exploration and understanding. Fungi comprise a minuscule part of the human microbiome called the mycobiome. Mycobiome is ubiquitous in the human body but a weakened immune system offers a leeway space for fungi to showcase its virulence. The role of mycobiome as a colonizer, facilitator, or driver of carcinogenesis is still ambiguous. Reactivating the mycobiome that undergoes collateral damage associated with cancer treatment can be watershed event in cancer research. The coordinated, virulent, non-virulent behavior of the fungi once they reach a critical density must be hacked, considering its diagnostic, prognostic and therapeutic implications in cancer. This review highlights the diversity of the mycobiome and its potential role in oral cancer.

## 1 Introduction

Oral squamous cell carcinoma (OSCC) is a well-recognized malignant neoplasm, responsible for more than 90% of malignancies of the head and neck region (1). The main factors that contribute to the development of OSCC are alcohol and tobacco (2). Oral potentially malignant disorders (OPMD) are a group of heterogeneous lesions associated with the risk of transformation into OSCC (3). Malignant transformation across all OPMD groups is about 8% and it is generally related to genetic, geographic, and lifestyle factors (4).

Several studies using animal models have indicated a potential causal relationship between the microbiome and the development of cancer. These findings have demonstrated that the microbiome can influence cancer progression through a variety of processes, which include the production of chemical metabolites implicated in carcinogenesis, inducing DNA damage, and regulating inflammation (5). The oral cavity contains approximately 700 different species of bacteria and nurtures a diverse community of bacteria, fungi, viruses, and protozoa (6). These microorganisms have a beneficial and harmonious relationship, working together to prevent the entry and attachment of harmful pathogens in the oral cavity. Dysbiosis, an imbalance in the microbial community, disrupts the control of pathogenic microorganisms and leads to an abnormal inflammatory or immune response against commensals. Dysbiosis has been proposed as a significant factor in the development of cancer, contributing to tissue changes associated with the disease. It has also been identified as a potential “hallmark of cancer” (5, 7).

When one microbial community is knocked out of the body (bacteria), another (fungi) takes a lead, can flourish and cause illness. If the communities are undisturbed, the fungal inhabitants appear to be harmless or perhaps even beneficial. Tumors are areas where the immune system has no access, thus fungi can naturally grow and flourish expanding its territory.

In the present article, we provide a comprehensive overview of the relationship between the oral mycobiome and oral cancer. The prevailing evidence on the involvement of fungi in oral cancer and their impending ability to facilitate, initiate, or promote the disease through different mechanisms is discussed.

## 2 OPMD and oral cancer: epidemiology and risk factors

OPMD, such as leukoplakia and erythroplakia, have a risk of progressing to OSCC. They may present a variety of characteristics, such as a change in the color of the mucosa (i.e., white, red, or a mixture of white and red), change in size, change in morphology (i.e., smooth, corrugated, granular, verrucous, atrophic, and/or plaque) (3). Epithelial dysplasia in OPMD may be contingent upon architectural changes, exhibiting minimal or no cytological abnormalities. Moreover, while definitive features may be elusive, the amalgamation of molecular, clinical, and microscopic characteristics contributes to an increased risk of developing an OSCC (8).

OSCC stands as a prominent malignancy in the head and neck region, originating specifically from the lips, oral cavity, and oropharynx (9). The worldwide prevalence of OSCC has been escalating, notably in regions such as Southeast and South Asia, Western and Eastern Europe, the Pacific, the Caribbean, and Latin America (10, 11). Oral and oropharyngeal cancer pose a significant global health challenge, with an annual incidence of 476,000 cases and an unfortunate mortality rate of approximately half of those affected (12). In 2023, it was projected that there would be 54,540 new cases of oral and oropharyngeal cancer in the United States (13). According to GLOBOCAN 2020, India holds the third position worldwide in terms of cancer incidence, and projections indicate a substantial increase to 2.08 million cases by 2040, representing a 57% rise from 2020 (12). In India, the oral cavity and pharynx are potential sites for cancer, accounting for a burden of 198,438 cases (14).

Alcohol and tobacco are some of the risk factors for OSCC (9). Tobacco impairs oral immunity, favoring gingivitis/periodontitis and oral cancer (15). *Porphyromonas gingivalis* and *Fusobacterium nucleatum* are widely studied bacteria known for their association with the onset of gingivitis and periodontitis. Additionally, these bacteria have been identified as contributors to carcinogenesis in mice (16). Furthermore, *P. gingivalis* has been suggested to be involved in oral-digestive cancer (17). Alcohol, specifically ethanol, serves as both a local and systemic risk factor, increasing the permeability of the oral mucosa, causing the dissolution of epithelial lipids, inducing epithelial atrophy, and disrupting DNA synthesis and repair. Chronic alcohol use exerts genotoxic and mutagenic effects, along with a potential decrease in both innate and acquired immunity (18). Human papillomavirus (HPV) and ultraviolet (UV) exposure represent additional risk factors for oral cancer (19, 20), accounting for 2%–8% of OSCC (21).

## 3 Relationship of oral and systemic fungi with oral cancer

### 3.1 Occurrence of fungi in the oral cavity in states of health and disease

The term “fungi” encompasses a diverse array of eukaryotic microbes that collectively constitute the human microbiota, referred to as “mycobiota”, coexisting harmoniously in virtually any anatomical location (22). Within the oral cavity, more than 75 genera, such as *Candida*, *Cladosporium*, *Aureobasidium*, and *Aspergillus*, contribute to this fungal community (23). Recent metagenomics studies have unveiled previously unidentified fungal species. Ghannoum et al. (23), in their molecular profiling study, identified a total of 85 species in the oral cavities of 20 healthy individuals, encompassing 11 non-culturable and 74 culturable fungi. Another study identified the cultivation of 101 different genera of fungi, with the number of species per individual ranging from nine to 23. Furthermore, using sequencing techniques, *Malassezia* sp. was identified as an additional pathogenic species commonly inhabiting the oral cavity (24). Similarly, a systematic review has documented the presence of *Candida* spp. and *Malassezia* spp. on mucous membranes, further emphasizing the diverse fungal composition within the oral environment (25).

The oral mycobiota exhibits remarkable diversity, primarily composed of organisms affiliated with the phylum *Ascomycota*, with *Candida* species standing out as a particularly prominent group. Culture-independent methods have unveiled an expanded spectrum that encompasses numerous general within the *Saccharomycetaceae* family. Notable members of this diverse group include, but not limited to, *C*. *albicans*, *Fusarium* species, *Pichia* species, *C*. *dubliniensis*, *Saccharomyces cerevisiae*, *C*. *rugose*, and *Hanseniaspora uvarum*. Collectively, these species represent the majority of fungi populating the oral cavity (26) (Table 1).

**Table 1:** Prevalence of oral mycobiome in healthy individuals.

Study	Most prevalent fungi
Urzúa et al. (27)	*Candida albicans* *Saccharomyces cerevisiae* *Candida dubliniensis* *Candida guillermondii* *Kluyveromyces lactis*
Ghannoum et al. (23)	*Candida* *Cladosporium* *Aureobasidium* *Saccharomycetales* *Aspergillus*
Dupuy et al. (24)	*Malassezia* *Aspergilllus* *Candida* *Pichia* *Cladosporium*
Monteiro-da-Silva et al. (28)	*Candida* *Rhodotorula* *Penicillium* *Aspergillus* *Cladosporium*
Peters et al. (29)	*Candida* *Aspergillus* *Pencillium* *Schizophyllum* *Rhodortorula*
Baraniya et al. (30)	*Malassezia* *Candida* *Cryptococcus* *Saccharomyces* *Trichoderma*
Khadija et al. (31)	*Candida* *Kluyveromyces* *Nakaseomyces* *Alternaria* *Aspergillus*

Fungi exhibit the characteristic ability to enhance cell density and promote the growth of hyphae, forming the structural framework for multispecies biofilms. Moreover, fungi stand out among eukaryotes due to their potent impact on the host immune system, exerting diverse immunological effects (32). Interactions between fungi and the host underscore the presence of a robust host immune mechanism (22, 32–34). In the realm of microbial ecology, fungi have been proposed as “keystone species”, playing a pivotal role in influencing the overall microbiota (35, 36).

*Candida* is known for its cross-kingdom relationships, its near-ubiquitous nature, and ease of cultivation (35, 36). Observations indicate that *C. albicans* is frequently detected species among individuals with OPMD and OSCC (37, 38). In a recent study assessing 100 patients with OSCC using biochemical methods, *Candida* species were identified in 74% of the samples, with *C. albicans* being the predominant species in 84% (38). Similarly, another study reported elevated levels of *C. albicans* in the saliva of individuals with head and neck squamous cell carcinoma (39). A study using molecular profiling techniques investigated the co-occurrence network of *C. albicans* in the oral rinse of cancer patients (40). The findings revealed variations in the prevalence of certain subtypes of *C. albicans*, with some exhibiting higher prevalence and others lower (40). In contrast, oral rinse samples from individuals without cancer showed a higher prevalence of *C. dubliniensis*, *Schizophyllum commune*, and organisms belonging to the *Agaricomycetes* class (40) (Table 2).

**Table 2:** Prevalence of oral mycobiome in oral cancer.

Study	Most prevalent fungi
Ayuningtyas et al. (41)	*Candida albicans*
Theofilou et al. (42)	*Candida spp*
İlhan B et al. (43)	*Candida albicans* *Candida glabarata* *Candida kruseii* *Candida tropicalis* *Candida parapsilosis*
Murugan et al. (44)	*Candida spp*
Fan et al. (45)	*Candida* *Aspergillus* *Alternaria* *Cryptococcus*
Tasso et al. (46)	*Candida albicans* *C. dubliniensis* *C. tropicalis* *C. glabrata* *C. parapsilosis*
Talapko et al. (47)	*Candida albicans*
Wang et al. (48)	*Candida albicans*
Wang et al. (49)	*Candida albicans*
He et al. (50)	*Verruconis gallopava* *Syncephalastrum racemosum* *Dimargaris cristalligena* *Lichteimia corymbifera* *Malassezia sympodialis*
Wang et al. (51)	*Candida albicans*

The progression of OSCC is marked by a significant increase in the presence of *Acremonium* and *Aspergillus*, both known for their detrimental health effects (52, 53). Conversely, *Morchella*, recognized for its potent inhibitory activity against harmful bacteria, experiences a noteworthy reduction in reduction in individuals with premalignant and malignant conditions. The polysaccharide FMP-1 derived from *Morchella esculenta* is attributed with prebiotic benefits and potential anti-tumor activity owing to its antioxidant capabilities (54, 55). The decline in *Morchella* levels suggests the potential emergence of invasive oral niches, heightening the risk of oral disorders. These findings offer a compelling perspective on the prevention and treatment of OSCC, underscoring the crucial roles played by distinct fungal species, including *Aspergillus fumigatus*, *C*. *tropicalis*, and *Acremonium exuviarum*, all labelled as “oncogenic fungi” (56). The mitochondrial toxicity of acrebol produced by *Acremonium exuviarum* (57), the ability of *A. fumigatus* to generate reactive oxygen species linked to oral cancer (58, 59), and the collaborative biofilm formation of *C. tropicalis* with bacteria such as *Escherichia coli* and *Serratia marcescens* (60) exemplify the significant health consequences demonstrated by these fungi.

According to Banerjee et al. (61), *Rhodotorula*, *Geotrichum*, and *Pneumocystis* were exclusively found in specimens collected from cancer patients, while *Fonsecaea* was absent in oral tissues from healthy participants. Intriguingly, *Fonsecaea* was present in cancer samples and tissues adjacent to malignancy from the same patients. Another study revealed that the only fungal species in tumor samples experiencing a significant decrease in matching non-tumor tissues was *Glomeromycota*. Notably, high T-stage tumor samples exhibited a higher fungal population compared to low T-stage samples (62). An important observation from the study by Perera et al. (63) is the overgrowth of *C. albicans* in OSCC tissue.

### 3.2 Role of fungi as drivers in oral cancer

It is well documented that candidiasis triggers type 3 responses initiated by activated IL-17-secreting cells These responses have been linked to tumor-promoting infiltrates of inflammatory cells and pose an antagonistic action to interferon-gamma (IFN-γ), a recognized anti-cancer defense mechanism (64). Conversely, candidalysin, a toxic epithelium-damaging substance produced by *Candida*, has been postulated to enhance tumor-promoting immunity (65–67). Despite this, the direct link between candidalysin and oral carcinogenesis remains unestablished. This recently identified *Candida* virulence factor has been demonstrated to activate additional oncogenic pathways within epithelial elements, involving the epidermal growth factor receptor/mitogen-activated protein kinase axis (42, 68, 69).

In a study on pancreatic cancer, the mannose-binding lectin, which binds specifically to the glycans on the fungal wall, was activated mainly by the *Malassezia* genus, showing potential for tumor formation (70). On the other hand, the presence of *Malassezia* sp. in OSCC is associated with a favorable prognosis (71).

While the oral mycobiome has demonstrated carcinogenic implications, substantial proof is currently lacking to establish whether these findings can be replicated for oral epithelial dysplasia. An alternative explanation posits that while these occurrences do exist in oral cancer growth, the associated changes may be insufficient to solely induce dysplastic alterations in oral epithelial tissue, serving more as accelerators rather that primary causes (72).

To mimic experimental oral carcinogenesis development, 4-nitrioquinolone-1-oxide (4NQO) has been used. This compound induces mutagenic changes in DNA similar to those triggered by tobacco (73). Live *C. albicans* has been found to enhance the migration potential, induce matrix metalloproteinase, and release associated metabolites via epithelial-mesenchymal shift, along with the upregulation of metastasis-associated genes (74). The same study explored the capacity of *Candida* to facilitate sequential cancer in 4NQO mice (74). These pre-neoplastic growths are supported by dysbiosis in the oral mycobiota, allowing them to progress into cancer (75). Antifungal therapy alone proves inadequate in treating the affected area and addressing this complex process; hence, the lesion should be treated as an OPMD (76).

Data from an exploratory study indicated that OSCC patients with *Candida* may experience lesser overall survival, serving as indirect proof for the potential tumor-promoting actions of mycobiome dysbiosis (71). In a recent metaproteomic analysis, a total of 196 fungal proteins demonstrated significant changes in abundance across different forms of OSCC. Most of the detected fungi had not been thoroughly investigated, and researchers were unaware of their toxicity or lethal associations in the oral cavity of humans (50). However, other fungi, including *Verruconis gallopava* (77), *Dimargaris cristalligena* (78, 79), and *Syncephalastrum racemosum* (80), were found to be more frequently present in OSCC lesions and linked to opportunistic infections.

Additionally, other well-known fungi species such as *Paracoccidioides brasiliensis*, *Malassezia sympodialis*, and *Lichteimia corymbifera* were detected. In an investigation comparing tissue from tumors with non-tumor controls, *L. corymbifera* showed an association with OSCC of the tongue (81). *P. brasiliensis*, a fungal yeast causing paracoccidioidomycosis leading to oral manifestations, has been associated with OSCC (82). Coexistence of oral paracoccidioidomycosis and OSCC has been reported (83), suggesting a potential role of fungi in cancer development, possibly due to continuous stimulation of epithelial cells increasing susceptibility to malignant transformation (84). Nonetheless, this association is not confirmed due to the limited number of experimental and clinical studies investigating this effect.

## 4 Cross-realm alliance: fungi and other microbes

**Figure 1:**
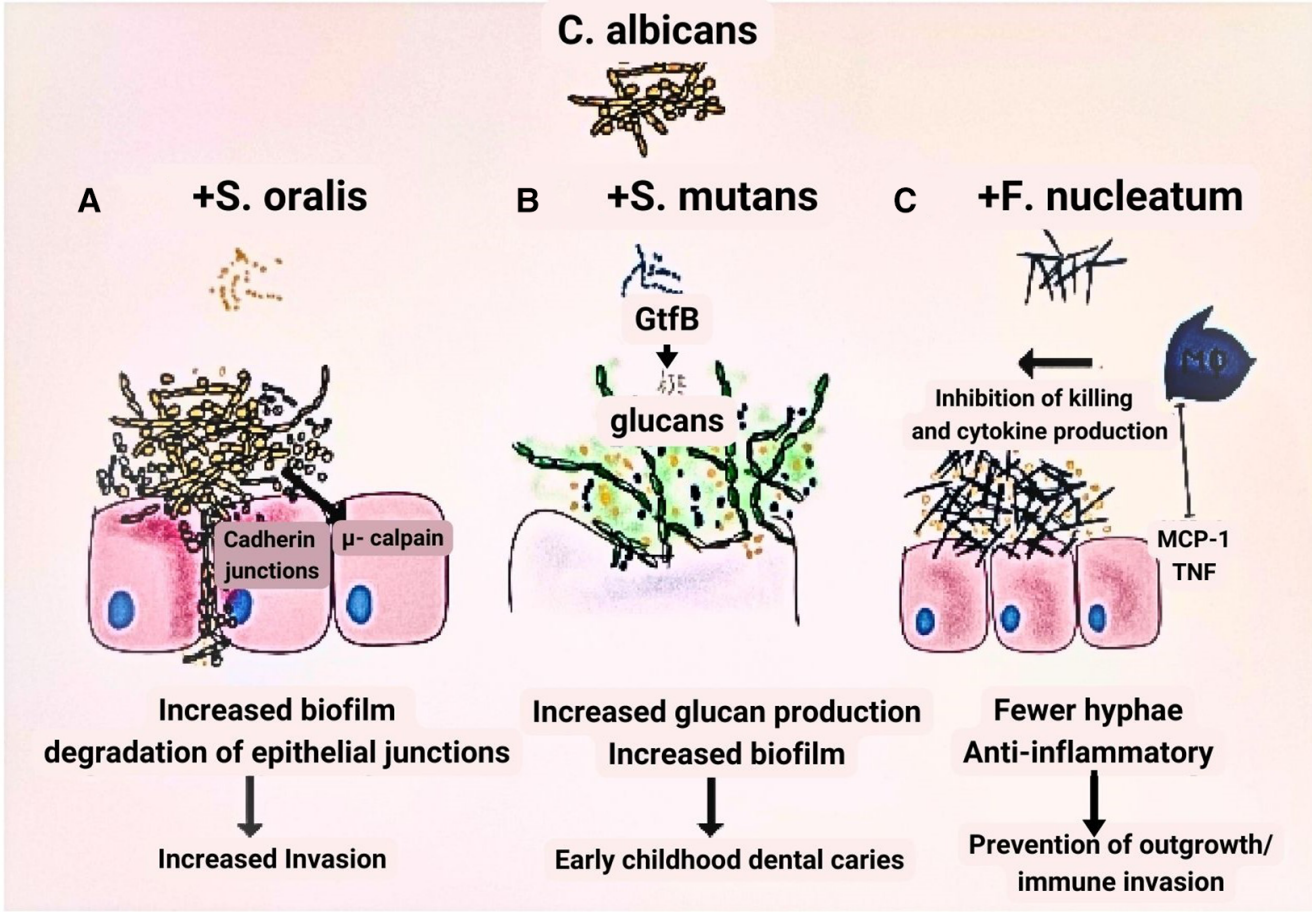
Model of fungal-bacteria relationship. (**A**) In contrast to when each species is grown independently, *Streptococcus oralis* and *Candida albicans* grow to a greater number when the two are combined in a biofilm. (**B**) The growth of glucan is one of the factors contributing to the virulence of *S. mutans* that is facilitated by the inclusion of *C. albicans. C. albicans* and *S. mutans* progress more densely than when cultured alone. (**C**) Growing *C. albicans* in combination with *Fusobacterium nucleatum* considerably reduces its capacity to produce hyphae.

The ability of *C. albicans* to form alliances with *F. nucleatum*, circumventing host defenses (85), and its interaction with *Streptococcus mutans* (86, 87), or *S. oralis* (88, 89) to exacerbate oral candidiasis or dental caries, respectively, underscores the importance of inter-kingdom associations in the development of polymicrobial conditions widely recognized today (72). Some authors propose that, as new research methods advance, the relationship between *C. albicans* and bacteria may emerge as a model for understanding fungal-bacterial interactions in the oral cavity (72) (Figure 1).

Soluble factors released by biofilms of *C. albicans* and *S. aureus* have been shown to induce variations in the expression of cell cycle genes and proto-oncogenes in both malignant and normal oral epithelial cell lines. This reveals how biofilm metabolites can influence gene expression and tumor cell survival based on cell-specific characteristics (90). In a study by Bertolini et al. (91) using animal models to target *Enterococcus faecalis* with antibiotics, the post-chemotherapy pathological effects of *C. albicans* infection on oral microorganisms were examined, uncovering the role of bacterial community changes in influencing the virulence of *C. albicans* in oropharyngeal candidiasis. Interactions among polymicrobial communities, particularly biofilm formation involving *C. albicans*, *Actinomyces naeslundii*, and *S. mutans*, were found to significantly enhance the adhesion of OSCC cells to extracellular matrix (ECM) and elevate the release of pro-inflammatory cytokines (92).

## 5 Role of the mycobiota in oral cancer

### 5.1 Inflammatory responses

**Figure 2:**
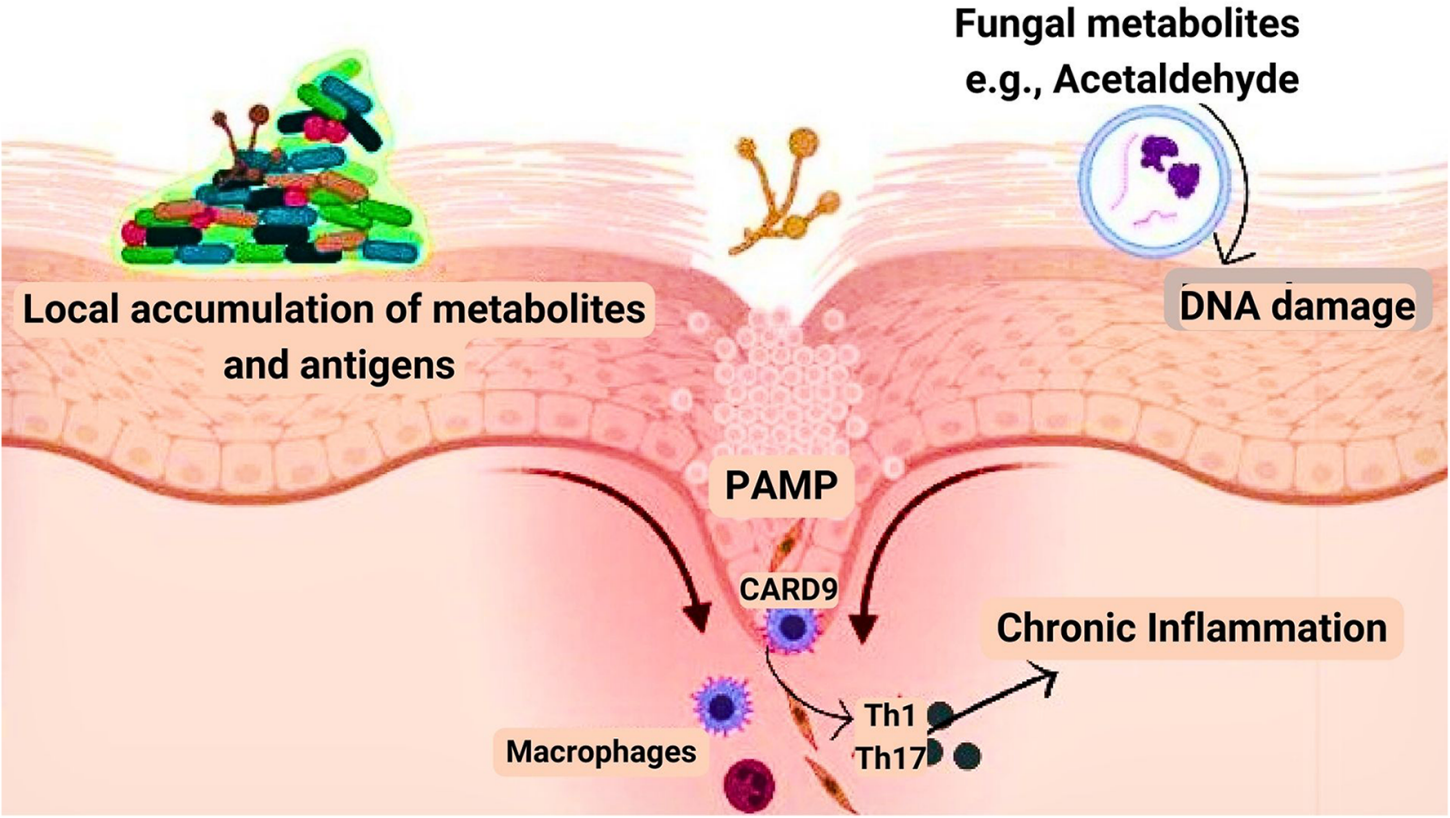
Mechanism by which fungi aid in cancer development. Pathogen-associated molecular patterns (PAMP), biofilm formation, and fungal metabolites such as acetaldehyde are the three major components involved in the development of cancer.

Several inflammatory diseases including ulcerative colitis, inflammatory bowel disease, and pancreatitis have been linked to cancer due to the inflammatory response elicited (93). The robust immune response elicited by fungal infection raises the possibility of its involvement, increasing the risk of oral cancer (22). Pattern recognition receptors (PRR) recognize pathogen-associated molecular patterns (PAMP) composed of carbohydrates in the fungal cell wall during fungal invasion, which initiates signaling cascades across multiple pathways such as *MYD88* (myeloid differentiation primary response gene 88), *SYK-CARD9* (spleen tyrosine kinase-caspase recruitment domain 9), and *TRIF* (Toll/IL-1R domain-containing adaptor-inducing interferon) pathways. Following the activation of these pathways numerous signaling molecules are secreted, such as interleukin (IL)-1, IL-6, IL-12, IL-23, transforming growth factor (TGF), and interferon (IFN). These signaling molecules regulate the antifungal response of T helper cell 1 (Th1) and T helper cell 17 (Th17) along with phagocyte stimulation and neutrophil recruitment (94) (Figure 2).

### 5.2 Formation of oral biofilm

Microbial biofilms are significant in a wide range of medical disorders (95–97). Existing research indicates that bacterial and fungal biofilms are equally significant. Few studies have highlighted that fungus and bacteria can work together to create biofilms that aggravate inflammation (98). It has been established that the disease-causing bacterial agent *Fusobacterium nucleatum*, which resides in the oral cavity and digestive system, plays a role in the emergence of colorectal cancer (99). Moreover, it has been shown that *F. nucleatum* and *C. albicans* co-aggregate by interacting between genetic and morphological cellular elements (100).

Mukherjee et al. (62) performed inter-kingdom analysis for fungi and bacteria in 39 samples which included oral tongue cancer and non-tumor samples. *Lichtheimia*, a fungal species, has demonstrated a constructive relationship with bacterial species such as *Campylobacter*, *Porphyromonas*, and *Fusobacterium*. Earlier studies have highlighted the role of gut bacteria, especially *F. nucleatum*, in the development of colorectal cancer (101). This type of cancer exhibits molecular gene silencing through CpG island methylation that has been associated with *F. nucleatum* (102).

### 5.3 Metabolites of fungus origin

Few studies have shown that the alcohol dehydrogenase enzyme found in different *Candida* species enables them to produce acetaldehyde (103–105) that is highly toxic, mutagenic, and carcinogenic (106). For example, Asian subjects lacking aldehyde dehydrogenase-2, an enzyme that aids in the body's elimination of the compound by oxidizing it to acetate, have higher salivary acetaldehyde levels and a 10-fold greater chance of oral cancer (107).

The mutagenic properties of the metabolic product of *Candida* alcohol dehydrogenase enzyme, i.e., acetaldehyde raise its possible implication in carcinogenesis (64). *Candida* has been demonstrated to exhibit higher metabolic rates and acetaldehyde synthesis in oral cancer compared to healthy controls (108). Moreover, *Candida* has pronounced nitrosation potential due to the production of nitrosamine compounds that have a significant role in oral carcinogenesis (75).

**Figure 3:**
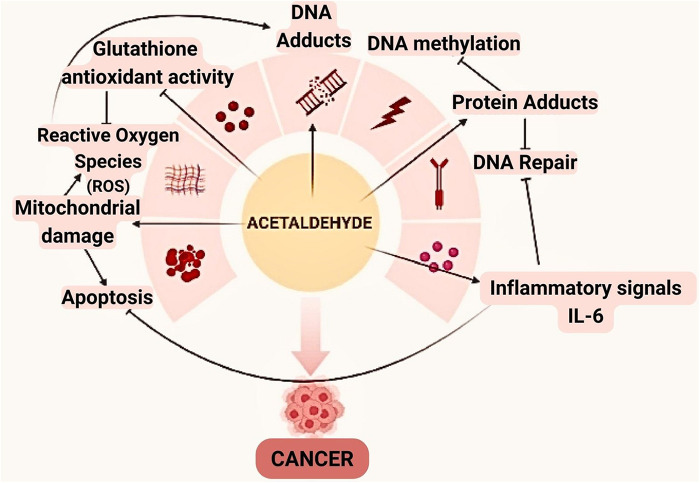
Acetaldehyde can be involved in the oral carcinogenesis through a variety of mechanisms. In addition to altering the framework and functionality of proteins and DNA by binding, this compound also reduces glutathione's antioxidant activity, which raises the amounts of reactive oxygen species (ROS).

Mitochondrial aldehyde dehydrogenase-2 is beneficial in eliminating acetaldehyde. However, dysbiosis with an increase in *Candida* may cause an elevated level in its production, especially in frequent drinkers (109, 110) (Figure 3).

## 6 Future directions

Cancer occurs when normal cells in the body expand, and divide exponentially. Certain immune cells are essential for tracing and destroying these tumor cells. This immune response is influenced, at least in part, by the microbiota. Immune cells have sensors that may identify specific microorganisms, triggering various reactions. A crucial component in the body's capacity to recognize fungi is a protein called dectin-1. It is found on the surface of some immune cells and functions as a transmembrane pattern-recognition receptor through its ability to bind β-glucan carbohydrates (111). It has been discovered to be associated with tumor growth and survival rates in both mouse and human breast tumors (112) and melanomas (54, 113). The effectiveness of cancer treatments, particularly radiotherapy, and its impact on the immune system, gut microbiota, and the other collateral damage it induces on the body may have a causal link with an overgrowth of specific fungi (113). This further emphasizes the potential relevance of the combination of standard chemotherapy or radiotherapy with therapies that regulate the oral microbiota.

**Figure 4:**
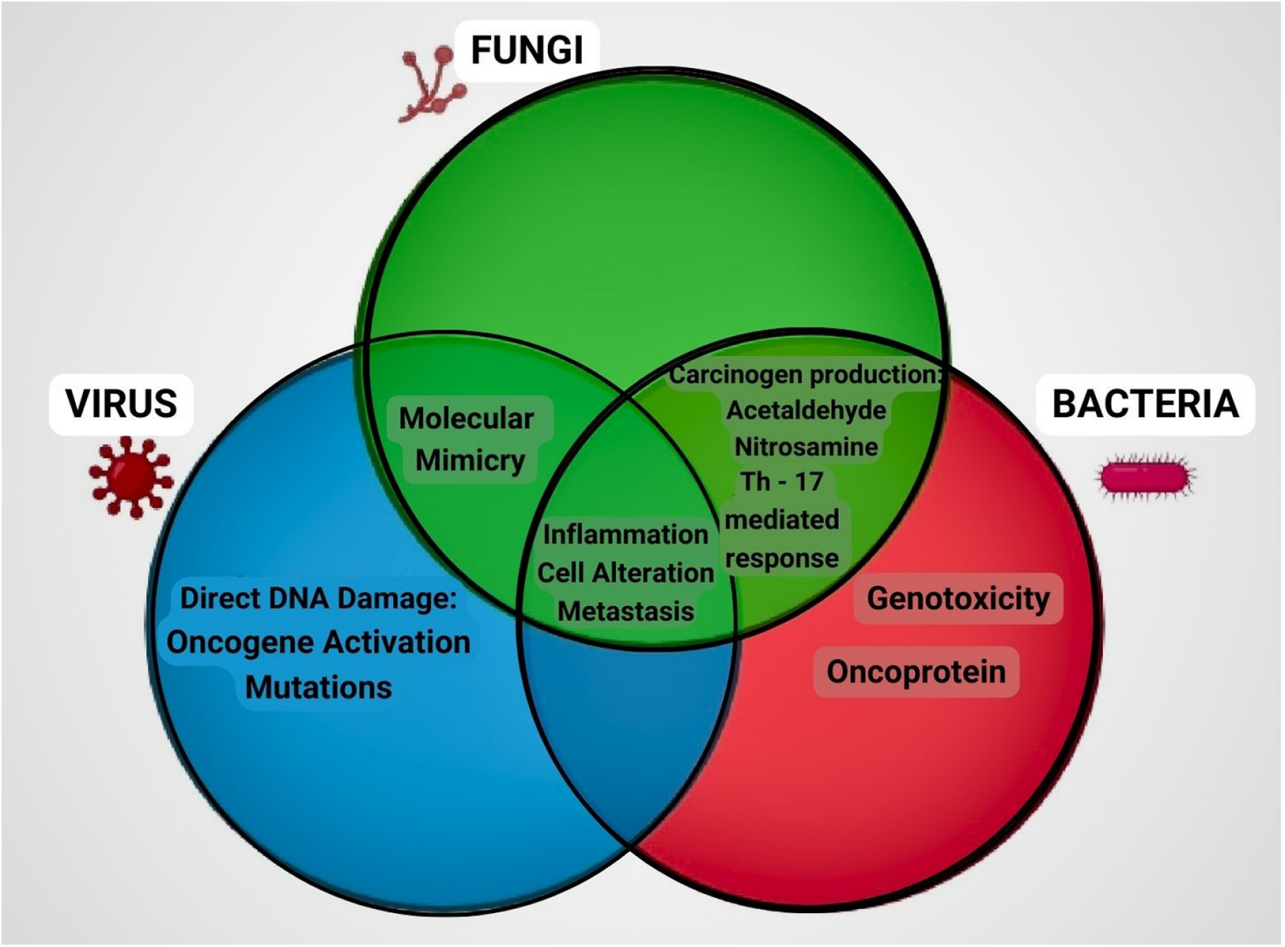
Role of fungi, bacteria and virus in carcinogenic process. Representation of the mechanisms involved in cancer progression by microorganisms through a Venn diagram. Fungi and bacteria produce metabolites like acetaldehyde and nitrosamine, induce inflammatory response through T helper cells. Fungi and virus mimic the molecular structure similar to that of the host thereby activating the immune system. Fungi, bacteria and virus all have the common mechanism of causing inflammation, cell alteration and metastasis which ultimately contributes to the development of cancer.

The discovery of a distinct fungal pattern that points to the involvement of fungal communities in tumor development highlights the need to attain traction and stretch the scope of microbiome research beyond bacterial communities. Earlier studies have illuminated the interplay between distinct microbial communities, implying that these joint communities contribute to dysbiosis or the preservation of a healthy microbiome (72). While there is a range of data on the role of *Candida* in oral carcinogenesis (37, 41, 114), other species such as *Aspergillus*, *Penicillium*, and *Malassezzia* require additional investigation (Figure 4). Overall, the study of the oral mycobiome is still in its infancy, and further research aiming at exploring the potential role of fungi in tuning and modulating the host immunity is essential to open up new possibilities for the prevention and adjunctive treatment of oral cancer.

## 7 Concluding remarks

The microbial communities, driven by their survival instincts within the host, employ cooperative evolutionary strategies that lead to the formation of robust biofilms. Fungi enhance their virulence through filamentation and increased secretion of aspartyl proteinases, strengthening their ability to invade the host (115). Conversely, bacteria develop antibacterial tolerance while residing under the protective fungal matrix umbrella. *C. albicans*, for instance, produces mucolytic enzymes that degrade the protective mucin layer of the epithelium, contributing to the formation of lesions, tumors, and other pathological conditions. This interkingdom cooperation significantly influences the host immune system (116).

The oral microbiome, comprising a diverse array of fungi, bacteria, and viruses living in biofilms, can pose a threat to immunocompromised oral cancer patients. While guidelines and protocols exist to control oral infections, they often rely more on clinical observations than robust evidence. Increased microbial load, especially in cancer patients, can be potentially fatal. Thus, maintaining good oral health and seeking regular treatment from oral health professionals is crucial. Although some components of the oral mycobiome can metabolize substances into carcinogens, their exact role in the development of oral cancer remains uncertain. For example, the involvement of mycobiota in alcohol metabolism can lead to the formation of acetaldehyde, a known carcinogen. The combination of poor oral hygiene, inadequate nutrition, and established risk factors such as alcohol and tobacco can amplify the risk of developing oral cancer.

The application of novel molecular methods is enabling the identification and understanding of the mycobiome's role in both healthy individuals and those immunosuppressed individuals. The inter-kingdom relationships between fungi and bacteria add another layer of complexity to this exploration. The detection of newly identified proteins closely associated with fungal overload allows for the assessment cancer treatment effectiveness and exploration of novel approaches. Thus, it is important to include the mycobiome—the concealed realm of potential biohackers—in research efforts. Decoding its molecular signature and evaluating its connection to oral cancer can have significant implications in prevention, diagnosis, and treatment, opening new horizons in cancer research.
